# Prevention of Acute Postoperative Pain in Breast Cancer: A Comparison between Opioids versus Ketamine in the Intraoperatory Analgesia

**DOI:** 10.1155/2021/3290289

**Published:** 2021-11-17

**Authors:** Mirian López, María Luz Padilla, Blas García, Javier Orozco, Ana María Rodilla

**Affiliations:** ^**1**^ Department of Anesthesia, Cartagena University Hospital, Murcia, Spain; ^**2**^ Department of Anesthesia, Almansa General Hospital, Albacete, Spain

## Abstract

**Background:**

Acute postoperative pain (APP) has a high incidence in breast surgery, and opioids are the most commonly used drugs for its management; however, they are not free from systemic side effects, which may increase comorbidity. In the past few years, opioid-free anaesthesia has been favoured with promising results.

**Methods:**

We conducted a descriptive study including 71 patients who underwent breast cancer surgery. The opioid group (*n* = 41) received fentanyl for induction, remifentanil for maintenance, and rescue morphine before waking up, whereas the ketamine group (*n* = 30) received a ketamine bolus for induction followed by continuous ketamine infusion during surgery. Later, the presence and intensity of pain were registered, using the Numeric Rating Scale (NRS 1–10) for pain, at different times in the recovery room, at 24 hours and at 3 months.

**Results:**

Administration of ketamine is more effective than opioid use for APP prevention in breast cancer surgery because the ketamine group presented with less pain than the opioid group (*p* < 0.05) at all measured times. When there was pain, patients in the ketamine group gave a lower score to its intensity (*p* < 0.05).

**Conclusions:**

Ketamine could reduce the incidence of APP in breast cancer surgery, compared to opioids.

## 1. Introduction

APP has an incidence of 77–86% depending on the type of surgery, the analgesia received, and the type of patient [1]. It has systemic consequences that increase comorbidity, besides posing a risk of becoming chronic (up to 13%), if not treated properly [2].

There are different drugs used for APP control, with opioids being the drug of choice [3]. However, they have many side effects, among which postoperative nausea and vomiting (PONV) and respiratory effects stand out, the former due to their frequency and the latter due to their clinical consequences [4–6].

With the purpose of improving analgesia while reducing the side effects of opioids, multimodal analgesia appeared, which includes opioid-less anaesthesia (OLA) and opioid-free anaesthesia (OFA) [7–17]. The patients who benefit most from this mainstream are those for which opioid use presents higher comorbidity, such as patients at risk of PONV, obese patients, patients with a chronic pulmonary disease, or patients undergoing cancer surgery in which opioid administration has been associated with tumour progression [18]. Animal and in vitro studies support the role of opioids both in immunosuppression and in tumour angiogenesis, and although currently there is no evidence in humans, there are ongoing studies pending completion [19–22]. On the other hand, APP secondary to surgery causes an immunosuppression state (with decreased NK cells and T lymphocytes); therefore, the type of analgesia administered for APP control may influence tumour development, and consequently, the patient's prognosis [23].

Taking into account the foregoing, we chose breast cancer surgery for our study because it is the most frequent cancer in women worldwide where surgery still plays a crucial role, with the incidence of APP being up to 67% [24] and with more predisposition to PONV due to the young age of women, for whom opioid removal may be beneficial. Thus, we proposed based on the analgesic technique on ketamine use.

## 2. Materials and Methods

This was a single-center retrospective cohort study. The study protocol adhered to the ethical guidelines of the 1975 Declaration of Helsinki, and the study was approved by the institutional review board of our institution. All patients provided informed consent prior to the participation in the study.

All numerical were recorded as mean, standard deviation, minimum, and maximum. And, qualitative variables were expressed as frequencies and percentages.

For hypothesis testing with continuous variables, first, sample normality was assessed and the conditions for homogeneity of variances. Means were compared by using either Student's *t*-distribution for two factors or ANOVA when there were more than 2 factors, also applying Tukey's test to find differences among subgroups when variable distribution was normal.

To compare qualitative variables, we used chi-square test and Fisher's exact test. The strength of association between qualitative variables was measured by calculating the corrected typified residuals. To study the relationship between continuous variables, we used Pearson's correlation coefficient in order to see the linear correlation or the Kendall rank correlation coefficient for the rest of possible disruptions. We used a repeated measures ANOVA model to see the effects of anaesthesia on the subjects for variables measured at more than two different times.

The odds ratio (OR) was established with a 95% confidence interval (CI) for opioid or ketamine use, the main dependent variable being the presence of APP. Besides, there are other covariates which may have an impact on APP, and therefore, they were considered as potential confounders in the binary logistic regression model. In order to calculate the number of independent variables which could be included in the multivariate analysis, Peduzzi's criteria were used.

The entire statistical analysis was performed by using the software IBM SPSS Statistics v20, and those differences reaching a value of *p* <0.05 were considered statistically significant.

### 2.1. Study Development

We conducted the study at the Cartagena University Hospital (Murcia, Spain) on 71 patients scheduled for breast cancer surgery. Patients were recruited during the preanaesthetic consultation.

**Figure 1:**
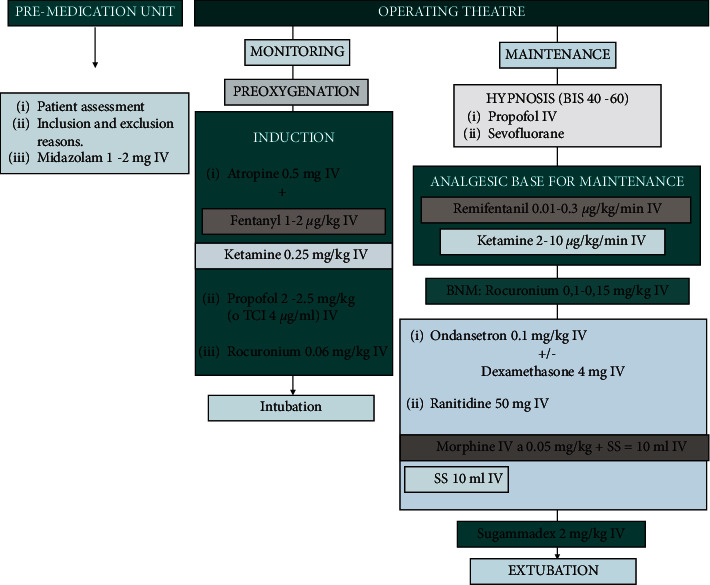
Anesthetic technique options.

On the day of the surgery, into the premedication unit, the main researcher assigned a personal identification number to the patient (which was recorded in the patient's data collection sheet). The anaesthesiologist responsible for the patient assigns the patient to the opioid or the ketamine group as the analgesic basis, in accordance with the usual practice (Figure 1).

In the operating theatre, the anaesthesiologist chose to induce anaesthesia with a bolus of 1-2 *μ*g/kg of fentanyl in group 1 or 0.25 mg/kg of ketamine in group 2. Then, patients received 2–2.5 mg/kg of propofol and 0.6 mg/kg of rocuronium bromide, intubating and thus connecting to mechanical ventilation. The adequate hypnosis level for surgery was induced either with propofol or with sevoflurane, and group 1 was administered with a continuous infusion of remifentanil at 0.01–0.3 *μ*g/kg/min, whereas group 2 was administered with an intravenous perfusion of ketamine at 2–10 *μ*g/kg/min as the analgesic base for maintenance during surgery.

During the final stage of surgery, gastric protection was provided with ranitidine and PONV prophylaxis performed with ondansetron and optionally a bolus of 4 mg of dexamethasone. Moreover, 1 g of paracetamol and NSAIDs were added as analgesia. For the opioid group, the anaesthesiologist added a bolus of 0.05 mg/kg of morphine.

Once extubated, the presence of immediate postoperative pain and its intensity (by a blinded observer, who was part of the nursing personnel and who did not know the group to which the patient had been assigned) was registered. Other variables were also registered: anaesthesia time, respiratory complications, alterations of consciousness, and the subjective quality of extubation and awakening.

In the recovery room, the nursing staff (blinded assistant) assessed the presence and severity of pain by using the NRS at 10, 60, and 90 min, administering rescue analgesia with NSAIDs for NRS <4 or morphine (0.1 mg/kg) for NRS ≥4.

At 24 h, the main researcher examined the need for rescue analgesia since discharge from the recovery room and applied the NRS score again. Lastly, at 3 months, the main researcher made a phone call to examine the presence of pain, thus concluding data collection.

## 3. Results and Discussion

### 3.1. Results

**Figure 2:**
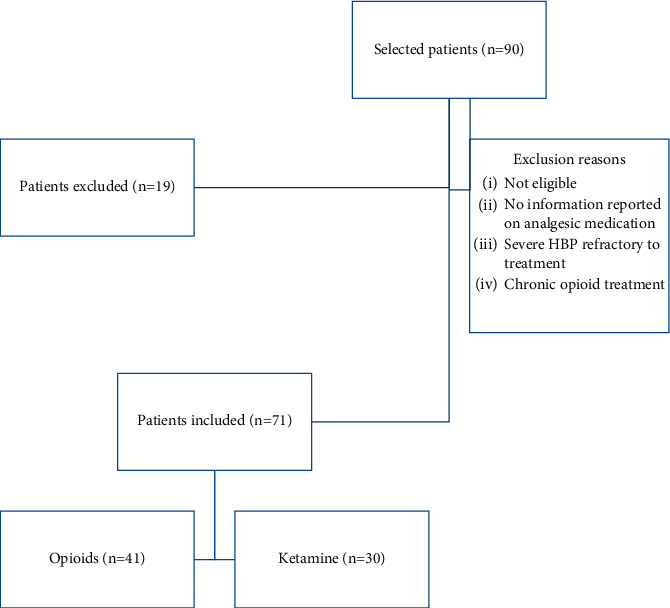
Flowchart showing the participant inclusion and exclusion process.

The sample in our study was made up of 71 women. Of the 90 patients recruited, 41 valid cases were for the opioid group and 30 for the ketamine group (Figure 2). The mean age in the opioid group was 60.59 years, and 53.93 in the ketamine group. No statistical significance was found regarding smoking habits, ASA classification, tumor stage at the time of surgery, prior administration of adjuvant therapy (chemotherapy or radiotherapy), and the type of surgical procedure performed or its duration. The type of anaesthesia was similar, and in both groups, no delay in discharge was observed (*p* > 0.05) (Table 1).

#### 3.1.1. Results of the Main Variables (Figure 3)

After 10 min in the recovery room, 68.3% of the patients in the opioid group had pain, in comparison with 26.7% of the patients in the ketamine group (*p* ≤ 0.001) (Table 2). In univariate analysis, ketamine was associated with a lower probability of pain at 10 min after extubation (OR 0.169, 95% CI 0.06–0.47, *p* ≤ 0.001) (Table 3), thus reducing by 83.1% the risk of having pain in comparison with the opioid group (Table 3).

At 60 min, 73.2% of the patients in the opioid group had pain in comparison with 36.7% in the ketamine group (*p* = 0.002) (Table 2). In the univariate analysis, IV ketamine was associated with a lower probability of pain at 60 min after extubation (OR 0.212, 95% CI 0.077–0.585, *p* = 0.003) (Table 3), thus reducing by 78.8% the risk of having pain.

At 90 min after extubation, 63.4% of patients in the opioid group had pain compared to only 23.3% of cases in the ketamine group (*p* ≤ 0.001) (Table 2). In the univariate analysis, IV ketamine was associated with a lower probability of pain at 90 min after extubation (OR 0.176, 95% CI 0.061–0.506, *p* ≤ 0.001) (Table 3), thus reducing by 82.4% the risk of having pain in comparison with the opioid group.

On the following day at the general ward, 56.1% of the patients treated with opioids, presented with pain compared to 3.3% of patients treated with ketamine (*p* ≤ 0.001) (Table 2). In the univariate analysis, IV ketamine was associated with a lower probability of pain at 24 h after hospitalization (OR 0.027, 95% CI 0.003–0.217, *p* ≤ 0.001) (Table 3), thus reducing by 97.3% the risk of having pain.

Finally, at 3 months, 4.9% of the patients treated with opioids, presented with pain compared to 3.3% of patients treated with ketamine (*p=*0.239) (Table 2).

This study also registered other variables that may have influenced APP in breast cancer surgery, regardless of the group to which the patient belong to (opioids/ketamine, age, BMI, lymphadenectomy performance, and type of hypnotic and corticoids). In accordance with the multivariate analysis, the administration of ketamine was associated with a lower probability of pain at 10 min after extubation (OR 0.144, 95% CI 0.043–0.477, *p* = 0.002), at 60 minutes after extubation (OR 0.197, 95% CI 0.062–0.623, *p* = 0.006), at 90 min after extubation (OR 0.126, 95% CI 0.037–0.431, *p* ≤ 0.001), and at 24 h after hospitalization (OR 0.008, 95% CI 0.001–0.098, *p* ≤ 0.001) (Table 3). Moreover, ketamine administration is the most influencing variable on APP according to this analysis, at all the times measured, with a chi-square score of 12.009 at 10 min (*p* ≤ 0.001), 9.461 at 60 min (*p* = 0.002), 11.188 at 90 min (*p* ≤ 0.001), and 21.554 at 24 h after hospitalization (*p* ≤ 0.001). Furthermore, a lower BMI was associated with higher APP at 10 min, and younger age was associated with higher APP at 24 h after hospitalization. No significant differences were found for the rest of variables, including corticosteroids.

The intensity of APP was significantly lower in the group of patients who received ketamine than in the opioid group at 10 min, 60 min, 90 min, and 24 h after hospitalization (1.07 vs. 2.61, *p* = 0.006; 1.37 vs. 3.29, *p* = 0.003; 0.73 vs. 1.46 *p* = 0.498; 0.13 vs. 1.88 *p* ≤ 0.001). Regarding the need for rescue analgesia during recovery, it was necessary in both groups. However, in the general ward, we did find significant differences because the opioid group required more rescue analgesia (29.3% vs. 3.3%, *p* = 0.005) (Table 2).

The quality of awakening, the presence of complications during extubation, or in the immediate postoperative period (PONV, alterations of consciousness, and anxiety) was similar for both groups. During surgery, there were indeed significant differences in HR; however, it is worth noting that all hemodynamic values were within normal limits, so they were not considered as having detrimental clinical effects. Additionally, hemodynamic trends were monitored for each group, and we observed that SBP, DBP, and HR were significantly lower (*p* < 0.05) in the opioid group after the administration of such drugs in the operating theatre, an effect that is attributed to these drugs according to the literature. The ketamine group, however, showed no significant differences.

### 3.2. Discussion

The incidence of severe APP after breast surgery is about 61–67%, depending on surgical invasiveness [24]. It has been proven that inadequate treatment of APP is one of the main risk factors associated with chronic postsurgical pain (25–80% after breast surgery) [25]. Therefore, preventive analgesia is the best strategy to reduce this risk [26]. In this vein, regional anaesthesia is considered a good alternative to achieve good pain management, with decreased opioid consumption. Nevertheless, given that it is an invasive technique, it is not free from risks and requires training [27].

On the other hand, residual neuropathic pain is present in 20% to 68% of patients with breast cancer [28, 29]. In this type of pain, NMDA receptor antagonists, such as ketamine, have been proved to reduce it [30–32].

The use of ketamine was limited due to the side effects associated; however, a few years ago, this drug was rescued, and it has made its way in new lines of research which evidence its excellent analgesic power [33–37].

In 2018, Cochrane conducted one of the most comprehensive reviews (*n* = 8341) observing a reduction in postoperative opioid consumption of up to 19%, a reduction in pain score, an increase in times for rescue analgesia, and a reduction of the area of hyperalgesia. It concluded that ketamine may be particularly effective in surgeries causing moderate or severe pain. However, it was a nonstratified analysis (regardless of the type of intervention, dose or timing of ketamine administration). [38].

Another study conducted by Mulier et al. in 2019 on 193 DIEP flat reconstructions confirms the benefits of OFA, showing lower pain scores in analgesic scales, a reduction in opioid consumption and in PONV and, therefore, an improvement in recovery after surgery with the use of ketamine in low doses [14].

Therefore, it could be concluded that the aforementioned studies support the results obtained in this study, which shows that ketamine administration during the intraoperative period, instead of opioids, is more effective than opioids to prevent APP in patients undergoing breast cancer surgery, which ranges from minimally invasive to more aggressive surgeries, the latter entailing higher expected APP. In the first time measured, the ketamine group presented with a significantly decreased incidence of APP than the opioid group (*p* < 0.05). The intensity of pain for this group was also significantly lower (*p* ≤ 0.05). However, at 3 months, no significant differences were found between groups (*p* > 0.05).

The requirements for rescue analgesia at 24 h after surgery, mainly with NSAIDs, were lower in the ketamine group, with significant differences in comparison with the opioid group (3.3% vs. 29.3%, *p* = 0.005). It could be concluded that the opioid group had a higher need for rescue analgesia due to pain at 24 h after surgery. However, these differences are not so evident during the first few hours after surgery because during their stay in the recovery room, both groups required rescue analgesia with morphine in a similar manner (46.3% in the opioid group vs. 36.7% in the ketamine group, *p* = 0.635).

Regarding ketamine effects on ventilator weaning, our study showed no differences in comparison with the opioid group (*p* = 0.702), as reported by the studies published by Buchheitet al. [39].

Corticosteroids were administered only with the purpose of reducing the risk of PONV in this type of patients, they have been included in numerous OFA protocols, such as Mulier et al., and they may act as a confounding factor influencing the main variable, APP. Thus, a multivariate analysis was conducted to determine the influence of corticosteroids on APP, which ruled out such a relationship in our study.

In our study, no significant difference was observed between the groups regarding PONV, contrary to others [7, 11–14, 38, 40]. On the other hand, it can be concluded that both analgesic options provide hemodynamic safety and stability during the entire perioperative period.

Regarding alterations of consciousness, neither the latest Cochrane review nor our study showed significant differences regarding their occurrence in the ketamine or the opioid group in breast cancer surgery (*p* = 0.472), contrary to previous studies like the one published by Subramaniam et al. The incidence of agitation or delirium was not linked to the anaesthetic technique either (*p* = 0.130). In both groups, no delay in discharge was observed (*p* *=* 0.822).

One of the limitations of the study is the possible interference of opioid-associated hyperalgesia. Remifentanil is closely related to this paradoxical phenomenon, and the results could have been different with another type of opioid. Another limitation would be related to the morphine dose, which could have been insufficient to provide sufficient postoperative analgesia.

## 4. Conclusions

It can be concluded that both the presence of pain and its intensity were lower at all the times measured in the patients who received ketamine in comparison with the patients in the opioid group. Ketamine provides enough and safe analgesic effects in the field of breast cancer surgery.

The literature analysed supports the analgesic effect of ketamine in APP. However, the optimal moment for its administration and the initial and maintenance dose are still controversial. This study poses additional questions which may open other lines of research. A specific study should be designed to assess the duration of ketamine analgesia in APP, as well as to study the incidence of chronic postmastectomy pain after administering ketamine.

## Figures and Tables

**Figure 3:**
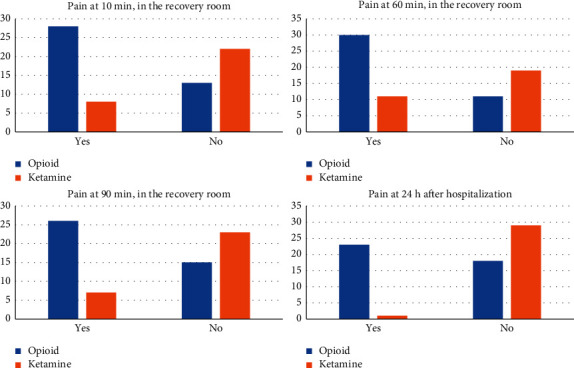
Number of patients of pain at different times (blue with opioid and green with ketamine).

**Table 1:** Results of variables analysed in the study, differentiated by group.

	Group opioid (*n* = 41)	Group ketamine (*n* = 30)	*p* value
Age (years)	60.59 ± 17.00	53.93 ± 17.62	0.113
Weight (kg)	69.95 ± 16.77	71.83 ± 17.40	0.648
Height(m)	1.60 ± 0.08	1.59 ± 0.07	0.780
BMI (kg/m^2^)	27.40 ± 5.98	27.89 ± 5.82	0.734
ASA I	3 (7.3%)	5 (16.7%)	0.249
ASA II	22 (53.7%)	18 (60%)	0.249
ASA III	16 (39%)	7 (23.3%)	0.249
Smoker	14 (34.1%)	13 (43.3%)	0.431
Mastectomy	12 (29.3%)	8 (26.7%)	0.599
Modified radical mastectomy	6 (14.6%)	4 (13.3%)	0.599
Quadrantectomy	12 (29.3%)	14 (46.7%)	0.599
Lumpectomy	9 (22%)	4 (13.3%)	0.599
Lumpectomy and reduction	1 (2.4%)	0 (0%)	0.599
Bilateral mastectomy	1 (2.4%)	0 (0%)	0.599
Surgery time (min)	98.22 ± 53.80	94.70 ± 39.71	0.763
Anesthesia time (min)	124.85 ± 55.37	121.80 ± 38.97	0.797
Time from end of analgesia to extubation (min)	27.76 ± 31.97	30.43 ± 24.42	0.702
Lymphadenectomy	14 (34.1%)	8 (26.7%)	0.501
SLNB	23 (56.1%)	18 (60%)	0.742
Stage of cancer IA	13 (31.7%)	14 (46.7%)	0.424
Stage of cancer IB	3 (7.3%)	0 (0%)	0.424
Stage of cancer IIA	8 (19.5%)	7 (23.3%)	0.424
Stage of cancer IIB	7 (17.1%)	2 (6.7%)	0.424
Stage of cancer IIIA	5 (12.2%)	3 (10%)	0.424
Stage of cancer IIIB	2 (4.9%)	3 (10%)	0.424
Stage of cancer IIIC	1 (2.4%)	1 (3.3%)	0.424
Stage of cancer IV	2 (4.9%)	0 (0%)	0.424
Radiotherapy	10 (24.4%)	6 (20%)	0.662
Chemotherapy	19 (46.3%)	9 (30%)	0.164
Sevoflurane	29 (70.7%)	20 (63.3%)	0.511
Propofol	12 (29.3%)	11 (36.7%)	0.511
Benzodiazepine	35 (85.4%)	28 (93.3%)	0.294
Corticosteroids	28 (68.3%)	27 (90%)	0.061
Alterations in the level of consciousness in recovery room	3 (7.3%)	1 (3.3%)	0.472
Agitation or delirium	3 (7.3%)	0 (0%)	0.130
Delay in discharge	1 (2.4%)	1 (3.2%)	0.822

Data expressed as *n* (%), mean ± standard deviation of patients within the group. ASA: ASA score. BMI: body mass index. kg: kilograms. m: meters. Smoker: more than one cigarette a day. SLNB: sentinel lymph node biopsy. *p* < 0.05. min: minutes. h: hours.

**Table 2:** APP and its intensity using the numerical rating scale (NRS) measured at different times in addition to the need for rescue analgesia during recovery and hospitalization.

	Opioid group (*n* = 41)	Ketamine group (*n* = 30)	*p* value
Pain after extubation	0 (0%)	0 (0%)	—
Pain at 10 min	28 (68.3%)	8 (26.7%)	0.001
Pain at 60 min	30 (73.2%)	11 (36.7%)	0.002
Pain at 60 min	26 (63.4%)	7 (23.3%)	0.001
Pain at 24 h after hospitalization	23 (56.1%)	1 (3.3%)	<0.001
Pain at 3 months	2 (4.9%)	1 (3.3%)	0.239
NRS after 10 minutes	2.61 ± 2.34	1.07 ± 2.20	0.006
NRS after 60 min	3.29 ± 2.86	1.37 ± 2.22	0.003
NRS after 90 min	1.46 ± 1.43	0.73 ± 1.64	0.049
NRS at 24 h after hospitalization	1.88 ± 2.25	0.13 ± 0.39	<0.001
Analgesia rescue in recovery room	19 (46.3%)	11 (36.7%)	0.635
Type of rescue in recovery room			
NSAIDs	5 (12.2%)	2 (6.7%)	0.635
Morphine	14 (34.1%)	9 (30%)	
Analgesia rescue at 24 h after hospitalization	12 (29.3%)	1 (3.3%)	0.005
Type of rescue at 24 h after hospitalization:			
NSAIDs	14 (34.1%)	3 (10%)	0.037
Morphine	1 (2.4%)	0 (0%)	

For the APP and for rescue analgesia data expressed as *n* and % of patients within the group. For the NRS, data expressed as mean ± standard deviation. Data expressed as *n* and % of patients within the group. *p* < 0.05. min: minutes. h: hours.

**Table 3:** First, univariate analysis, unadjusted binary logistic regression at different times, multivariate analysis, and adjusted binary logistic regression at different times.

	Odds ratio [95% CI]	*p* value	Chi^2^ score
Group (opioid/ketamine) at 10 min	0.169 [0.06–0.479]	0.001	—
Group (opioid/ketamine) at 60 min	0.212 [0.077–0.585]	0.003	—
Group (opioid/ketamine) at 90 min	0.176 [0.061–0.506]	0.001	—
Group (opioid/ketamine) at 24 h after hospitalization	0.027 [0.003–0.217]	0.001	—
Group (opioid/ketamine) at 10 min	0.144 [0.043–0.477]	0.002	12.009
Group (opioid/ketamine) at 60 min	0.197 [0.062–0.623]	0.006	9.461
Group (opioid/ketamine) at 90 min	0.126 [0.037–0.431]	0.001	11.188
Group (opioid/ketamine) at 24 h after hospitalization	0.008 [0.001–0.098]	0.001	21.554

*p* < 0.05. min: minutes. h: hours.

## Data Availability

The data used to support the findings of this study are available from the corresponding author upon request.
